# Investigation of Hydrogel Isolated from Seeds of *Ocimum basilicum* as Binder

**DOI:** 10.4103/0250-474X.56030

**Published:** 2009

**Authors:** A. V. Bhosale, S. Hardikar, A. A. Pathak, R. V. Sable

**Affiliations:** Poona District Education Association's Seth Govind Raghunath Sable College of Pharmacy, Saswad, Pune-412 301, India

**Keywords:** *Ocimum basilicum*, compressibility and compactibility, novel binder, hydrogel

## Abstract

Ayurvedic powders are widely used as therapeutic agents but most of them have unpleasant taste and large doses. One of the possible approach to overcome these drawbacks is to represent them in unit dosage form i.e. tablet dosage form. The purpose of this study is to elucidate and quantify the compressibility and compactibility of herbal granules prepared by using hydrogel isolated from whole seeds of *Ocimum basilicum* as a novel binder. The compressibility is the ability of the powder to deform under pressure and the compactibility is the ability of a powder to form coherent compacts. To test the functionality of novel excipients, Sonnergaard proved a simple linear model to confirm compactability, which is an uncomplicated tool for quantification. The tablets were compressed at increasing compression pressures and were evaluated for various mechanical properties. The linear relationship between specific crushing strength and compression pressure revealed the compactibility of the herbal granules and the linear relationship between porosity and logarithm of compression pressure revealed the compressible nature of the herbal granules according to the model developed by Sonnergaard. Thus the hydrogel isolated from whole seeds of *Ocimum basillicum* had potential as a granulating and binding agent.

In the traditional system of medicines e.g. Ayurveda, many of the remedies are in the form of powders. The dose of these powders is very large, many of them are difficult to swallow or give poor bioavailability owing to their poor wetting and many have pungent or unacceptable taste. It is proposed in Ayurveda that ideally dosage form should be pleasantly tasted and in a compact form[1]. One of the possible approaches to overcome these drawbacks of Ayurvedic powders is to present them in the form of a unit dosage form i.e. tablet. *Triphala* powder is widely used as anti diabetic and for eyesight improvement[2] in Indian population and has poor compressibility index (40%). Hence *Triphala* powder was selected as a model for investigation of binding and granulating properties of hydrogel isolated from whole seeds of *Ocimum basilicum.* Compressibility can be imparted by preparing granules following wet granulation technology and by using suitable granulating and binding agent[3]. In an attempt to avoid complexities associated with development of synthetic and semi synthetic excipients, an unexplored area of herbal products was tried. The whole seeds of *Ocimum basilicum* were authenticated by Agharkar Research Institute, Pune. The hydrogel[4] was isolated from whole seeds and used as a binding and granulating agent to prepare granules of *Triphala* powder. The seeds were soaked in deminerised water in a proportion 1:20 for 2 hrs. Then the whole mass was diluted with demineralised water, homogenised and centrifuged to isolate hydrogel from seeds.

Hydrogel was characterised for appearance, specific gravity and viscosity. Hydrogel was white, translucent and viscous liquid with specific gravity 1.013[5]. Viscosity of hydrogel was measured by using Brookfield viscometer (Model- DV II +) with spindle number 61. Viscosity of liquid decreased with increasing rate of shear. Thus hydrogel was shear thinning system. The results are reported in Table 1. It was also scanned for UV absorption. It absorbs UV radiation at 296 nm. This can be used as identification and quantification test of hydrogel as it obeys Beers Lamberts Law in the range 2 ppm to 16 ppm (fig. 1). About 25 g of seeds yielded 1.1 l of hydrogel of specifications reported in Table 1.

**Fig. 1:**
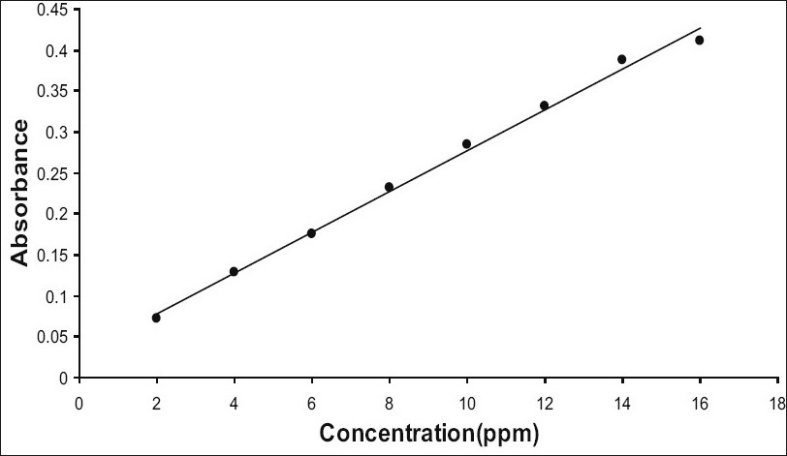
Calibration curve for hydrogel in the range 2 to 16 ppm. The equation of the line obtained was y= 0.0249x+0.0288 with r^2^ value of 0.9951.

**TABLE 1:** RESULTS OF MEASUREMENT OF VISCOSITY OF HYDROGEL

Speed of a spindle* (rpm)	% Torque	Viscosity (cp)
30	8.1	16.3
50	11.6	14
60	13.1	13.1
100	21.5	12.8

Cp is viscosity of hydrogel in centipoises.

* Spindle number 61 was used for viscosity measurement.

Hydrogel is liable to exhibit microbial contamination and stock solutions therefore should be preserved with a combination of methyl paraben and propyl paraben. It is most stable at pH 5 to 7. The bulk stock of hydrogel should be stored in airtight glass container and at cool place. Granules of *Triphala* powder were prepared by wet granulation technology adding sufficient quantity of hydrogel as binding and granulating agent[3]. The quantity of hydrogel of given specifications required was 3 gm to prepare the granules of 10 g *Triphala* powder. The granules were evaluated for particle size distribution, moisture content and flow properties[6,7]. Particle size distribution of granules was between 0.99 to 1.3 μ. Moisture content of granules was 0.07%, Hausner ratio was 0.149 and compressibility index was 13%. These results revealed that granules possessed necessary compression characteristics and they were suitable for tabletting. The granules were compressed on a mini rotary tablet press (Mini press II MT) with 8 mm diameter, circular and flat punches. Tablets were prepared at five different compression pressures (P) viz. 1, 2, 3, 4 and 5 Tons.

Thickness (h), weight (w) and diameter (d) of intact, ejected tablets were measured. Results are reported in Table 2 and are the means of ten measurements. The porosity of a tablet was calculated from tablet dimensions, weight of the tablet and particle density according to the Eqn. 1[8], ε= 1-4w/πd^2^hρ -1, where, ε is the porosity of a tablet, w is the weight of the tablet, d the diameter of the tablet, h the height of the tablet and ρ is the true density of the granules. The mechanical properties of tablets were determined[9,10]. Hardness or crushing strength for ten tablets was measured by tablet strength tester (Pfizer hardness tester).Results are reported in Table 2. Specific tensile strength (SCP) was calculated according to Eqn. 2[9], SCP = F/dh -2, where, F is the crushing strength or hardness of a tablet, d is the diameter of the tablet and h is the height of the tablet. The compaction properties of pharmaceutical materials are compressibility and compactibility. Compressibility is the ability of the powder to deform under pressure and the compactibility is the ability of the powder to form coherent compacts. According to Zhao *et al.*^[11]^ a simple relationship between lnP and porosity indicates compressibility of material (Eqn. 3). ε= -C*lnP+d -3.

**TABLE 2:** RESULTS OF MECHANICAL STRENGTH PARAMETERS OF TABLETS AT VARIOUS COMPRESSION PRESSURES

Compression Pressure (P, Tonnes)	Diameter* (d, cm)	Height* (h, cm±SD)	Weight* (W, g±SD)	Porosity (ε)	Hardness* (F, kg/cm^2^±SD)	Specific crushing strength (SCS)
1	0.8	0.50±0.03	0.305±0.02	0.241	1.2±0.09	2.98
2	0.8	0.44±0.45	0.290±0.01	0.183	1.9±0.56	5.35
3	0.8	0.42±0.44	0.290±0.01	0.151	3.2±0.07	9.35
4	0.8	0.41±0.01	0.284±0.02	0.124	3.9±0.15	12.02
5	0.8	0.40±0.02	0.286±0.01	0.107	4.7±0.16	14.65

* All values indicate mean±SD (n=10)

In fig. 2, porosity of a tablet was plotted against logarithm of compression pressure. A linear relationship was observed. This indicated the compressible nature of granules. The compactibility is more relevant and interesting from practical point of view. With the growing interest in the functionality of excipients there was a need for simple and standardised measure of compactibility. Sonnergaard[9] established a simple linear model for compactibility. He calculated specific crushing strength (SCS) and showed that the slope of the regression line SCP= Cp*P+b represents dimensionless compactibility parameter[9–10]. In fig. 3 specific crushing strength was plotted against compression pressure. A straight line was obtained which revealed the compactibility of granules. Since above relations are linear, the *Triphala* granules had optimum compressibility and compactibility when prepared by using hydrogel obtained from whole seeds of *Ocimum basilicum* as a novel binding and granulating agent.

**Fig. 2:**
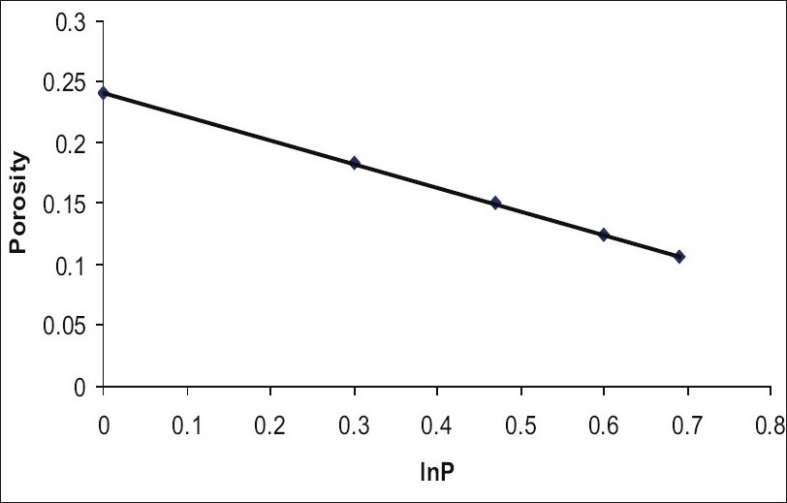
Linear relationship between porosity of tablets and log compression pressure. Compressibility of granules was confirmed by linear relationship between porosity of tablets and logarithm of compression pressure. The equation obtained was y = -0.1944x+0.2413 with r^2^ value of 0.9998.

**Fig. 3:**
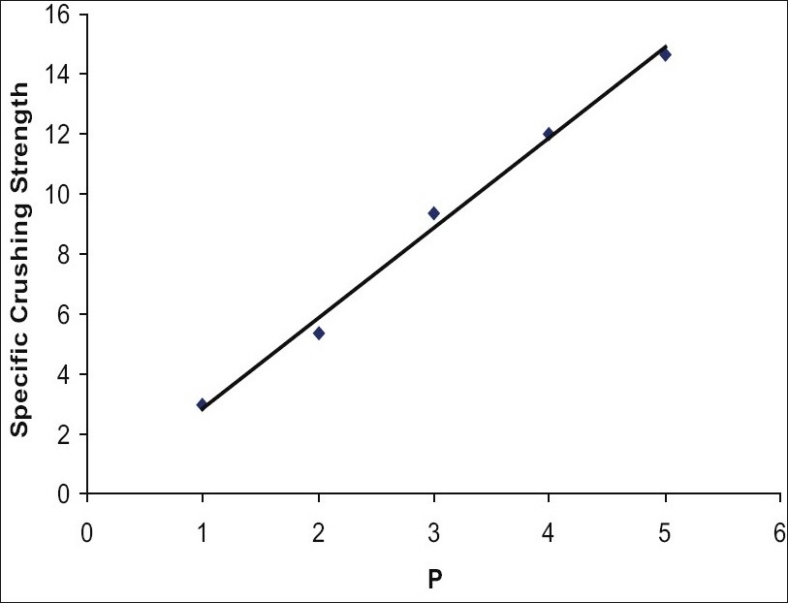
Linear relationship between specific crushing strength of tablets and compression pressure. Compactibility of granules was confirmed by linear relationship between specific crushing strength of tablets and compression pressure. The equation obtained was y = 3.001x-0.133 with r^2^ value of 0.9936.
